# 1186. Beta is Better: Impact of a Multifaceted Stewardship Initiative on the Sequential Timing of Beta-Lactam Administration

**DOI:** 10.1093/ofid/ofad500.1026

**Published:** 2023-11-27

**Authors:** Connor R Deri, Jennifer Schultheis, Jenny Shroba, John Boreyko, Rebekah Wrenn, Cynthia Keener, Rebekah W Moehring

**Affiliations:** Duke University, Durham, North Carolina; Duke University Hospital, Durham, North Carolina; Duke University Hospital, Durham, North Carolina; Duke Regional Hospital, Chapel Hill, North Carolina; Duke University, Durham, North Carolina; Duke University Hospital, Durham, North Carolina; Duke University, Durham, North Carolina

## Abstract

**Background:**

Appropriate antibiotic sequence (*i.e.* beta-lactam before vancomycin) may reduce early mortality in patients with bloodstream infections (BSIs). Herein, we describe the impact of a multifaceted stewardship initiative on the sequence of antibiotic administration across three hospitals.

**Methods:**

We performed a pre-post analysis describing the impact of a multifaceted stewardship initiative from March 1, 2021 – May 22, 2022 (pre) and May 31, 2022 – Feb 12, 2023 (post) on the sequence of antibiotic administrations in adults > 18 years old with at least one positive blood culture who received vancomycin and a beta-lactam (*i.e.* cefepime, meropenem, or piperacillin-tazobactam) across three hospitals: a major university hospital and two community hospitals. Our initiative consisted of 1) a health-system wide adult beta-lactam order panel combining load and maintenance doses for select beta-lactams (Figure 1) 2) nursing administration instructions facilitating the appropriate sequential order of antibiotic administration (*i.e.* beta-lactam before vancomycin) (Figure 2) and 3) system-wide education to physicians, pharmacists, and nurses. The rate of beta-lactam first administration was compared between groups. Beta-lactam first rates were further stratified by hospital and antibiotic administration locations. Chi-square tests were used to compare rates between groups.

Beta-Lactam Order Panel, Piperacillin-tazobactam

**Figure:**
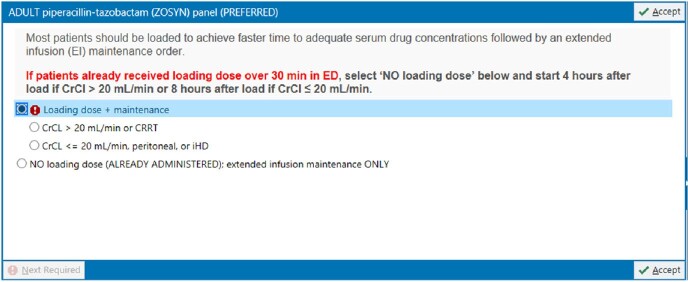

Beta-Lactam First: Nursing Administration Instructions, Cefepime

**Figure:**
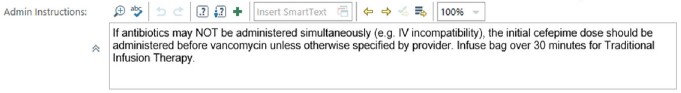

**Results:**

361 patients were included for analysis: 224 pre- and 137 post-intervention. Piperacillin-tazobactam was the most common beta-lactam among studied patients (71.5%, Table 1). Beta-lactam first rates were already high at baseline, then higher post-intervention compared to pre-intervention (96.4% vs 93.8%, p = 0.283) (Table 2).

**Table 1.**
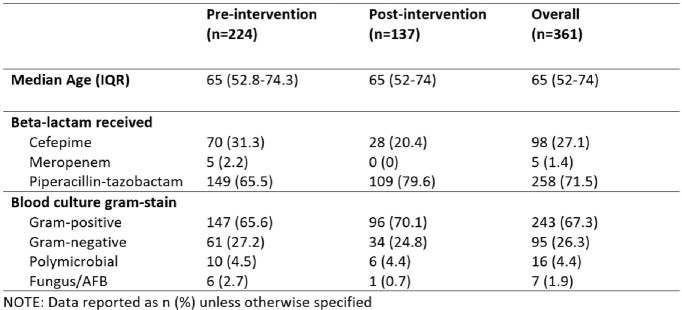
Receipt of Beta-Lactam Therapy in Adult Patients with Positive Blood Cultures

**Table 2.**
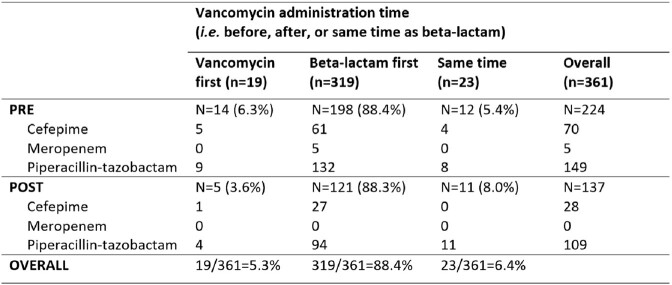
Sequence of Antibiotic Administration

**Conclusion:**

A multifaceted initiative to promote beta-lactam before vancomycin administration increased adherence to this preferred practice in our health system, though baseline rates were high. Clinical decision support paired with system-wide education involving key stakeholders can help standardize appropriate antibiotic sequence. Future study could evaluate impact on a larger scale, on patient outcomes, and evaluate sustainability of beta-lactam first practice.

**Disclosures:**

**Rebekah W. Moehring, MD, MPH, FIDSA, FSHEA**, UpToDate, Inc.: Author Royalties

